# Xylem and phloem phenology in co-occurring conifers exposed to drought

**DOI:** 10.1007/s00468-014-1026-x

**Published:** 2014-05-17

**Authors:** Irene Swidrak, Andreas Gruber, Walter Oberhuber

**Affiliations:** Institute of Botany, Leopold-Franzens-University of Innsbruck, Sternwartestrasse 15, 6020 Innsbruck, Austria

**Keywords:** Cambium, Drought, Intra-annual radial growth, Phloem formation, Xylogenesis

## Abstract

*****Key message***:**

**Variability in xylem and phloem phenology among years and species is caused by contrasting temperatures prevailing at the start of the growing season and species-specific sensitivity to drought.**

**Abstract:**

The focus of this study was to determine temporal dynamics of xylem and phloem formation in co-occurring deciduous and evergreen coniferous species in a dry inner Alpine environment (750 m a.s.l., Tyrol, Austria). By repeated micro-sampling of the stem, timing of key phenological dates of xylem and phloem formation was compared among mature *Pinus sylvestris*, *Larix decidua* and *Picea abies* during two consecutive years. Xylem formation in *P. sylvestris* started in mid and late April 2011 and 2012, respectively, and in both years about 2 week later in *P. abies* and *L. decidua*. Phloem formation preceded xylem formation on average by 3 week in *P. sylvestris*, and *c*. 5 week in *P. abies* and *L. decidua*. Based on modeled cell number increase, tracheid production peaked between early through late May 2011 and late May through mid-June 2012. Phloem formation culminated between late April and mid-May in 2011 and in late May 2012. Production of xylem and phloem cells continued for about 4 and 5–6 months, respectively. High variability in xylem increment among years and species is related to exogenous control by climatic factors and species-specific sensitivity to drought, respectively. On the other hand, production of phloem cells was quite homogenous and showed asymptotic decrease with respect to xylem cells indicating endogenous control. Results indicate that onset and culmination of xylem and phloem formation are controlled by early spring temperature, whereby strikingly advanced production of phloem compared to xylem cells suggests lower temperature requirement for initiation of the former.

## Introduction

The occurrence of phenological events in plant development such as bud break, leaf unfolding or flowering, is affected by climatic conditions (e.g., Chuine 2010) and several studies revealed shifts in phenological events in course of climate change (e.g., Menzel and Sparks 2006; Rutishauser et al. 2008). Cambial phenology, i.e., the determination of crucial phenological stages of xylem growth, i.e., onset, maximum and end of wood formation was monitored in deciduous and coniferous trees in recent years (e.g., Rossi et al. 2006b; Gruber et al. 2010; Michelot et al. 2012; Zhai et al. 2012). Knowledge of intra-annual dynamics of xylem formation is required to determine seasonal influence of meteorological factors on radial tree growth and evaluate species-specific response to global climate change. Cambial activity, however, also gives rise to phloem cells on the outer side and photoassimilates transported in the phloem sap are necessary during active radial stem growth as structural material, i.e., cell wall thickening and lignification and as source for metabolic energy (Maunoury-Danger et al. 2010). In contrast to numerous studies focusing on determination of seasonal dynamics of wood formation, reports on intra-annual development of xylem and/or phloem tissue in conifers of cold environments are scarce (Alfieri and Evert 1968, 1973; Antonova and Stasova 2006, 2008; Gričar et al. 2006, 2007, 2009; Gričar and Čufar 2008).

In most conifers, transport of nutrients (carbohydrates) and translocation of defensive and signaling compounds (metabolites, hormones) in living phloem cells occur for 1–2 years due to collapse of sieve cells. Only last layers of mature sieve cells overwinter and remain functional until new sieve cells differentiate in spring (Alfieri and Evert 1973; Evert 2006). On the other hand, water transport in dead xylem cells of coniferous trees (tracheids) can last for several decades (Trockenbrodt 1990; Larson 1994). A considerable disproportion was also found between xylem and phloem tissues, i.e., xylem mother cells divide more compared to phloem mother cells (Plomion et al. 2001; Evert 2006), whereby it was suggested that the ratio between xylem and phloem indicates tree vitality (Larson 1994; Gričar et al. 2009). Phloem formation was also reported to be less responsive to year-to-year variability of environmental factors compared to xylem formation (Alfieri and Evert 1968; Gričar et al. 2007; Gričar and Čufar 2008).

That cambium activity and xylem growth is highly responsive to temperature and plays a key role for cambial reactivation after winter dormancy was reported by several authors (e.g., Oribe et al. 2001; Deslauriers and Morin 2005; Gričar et al. 2006; Rossi et al. 2007; Lenz et al. 2013). Within the dry inner Alpine study area, results of Swidrak et al. (2011) also indicated that early spring temperature rather than soil water availability controls onset of radial stem growth in *Pinus sylvestris*. On the other hand, climate–growth relationships of coniferous tree species forming mixed stands at dry–mesic sites within the study area revealed different growth response of *P. sylvestris*, *Picea abies* and *Larix decidua* to precipitation (e.g., Pichler and Oberhuber 2007; Schuster and Oberhuber 2013a). While precipitation in April–May favored radial growth of *P. sylvestris*, annual increments of *P. abies* and *L. decidua* mainly depended on amount of rainfall in May–June. Different growth response to climate might be explained by a temporal shift in cambial activity, which can be elucidated by repeated cellular analyses of cambial dynamics during the growing season (e.g., Deslauriers et al. 2003; Rossi et al. 2006b). At the cellular level, drought can affect growth-related processes directly by restricting the turgor-driven process of cell enlargement and suppressing cambial cell division (Larcher 2003).

Simultaneous records of xylem and phloem development in co-occurring species could elucidate species-specific plasticity of cambial phenology in response to environmental stress. Therefore, the objectives of this study were (i) to compare intra-annual dynamics of xylem and phloem formation in a dry–mesic mixed-conifer forest where *P. sylvestris*, *L. decidua* and *P. abies* co-occur and (ii) to determine climate sensitivity of main phenological events of xylem and phloem formation, i.e., onset, time of maximum growth, end and duration during two consecutive years. We hypothesized that (i) contrasting intra-annual growth strategies are adopted by early (*P. sylvestris*, *L. decidua*) and late successional species (*P. abies*) and (ii) air temperature and water availability affect temporal dynamics of xylem formation, while phloem formation is unresponsive to these environmental factors but is endogenously controlled.

## Materials and methods

### Study area

The study site is part of a postglacial rock-slide area situated in the montane belt (*c.* 750 m a.s.l.) within the inner Alpine dry valley of the Inn River (Tyrol, Austria, 47°13′53″ N, 10°50′51″ E) and has a relatively continental climate with mean annual precipitation and temperature of 716 mm and 7.3 °C, respectively (long-term mean during 1911–2010 at Ötz, 812 m a.s.l., 5 km from the study area). The dominating plant community is an open Spring Heath-Pine wood (Erico-Pinetum typicum), whereby on scattered dry–mesic sites mixed stands composed of *P. sylvestris* (60 %), *L. decidua* (20 %) and *P. abies* (20 %) are developed. Shallow soils of protorendzina type, i.e., rendzic leptosols according to the FAO classification system (FAO 2006), are developed and consist of unconsolidated, coarse-textured materials with low water holding capacity (soil depth 10–20 cm). A thick moss layer dominates the understorey, indicating slightly moist conditions. A study plot where *P. sylvestris*, *L. decidua* and *P. abies* grow side by side was selected. Tree height and canopy coverage of the selected stand were 15–18 m and *c*. 70 %, respectively. The study site was slightly facing north (slope angle 5°).

### Xylem–phloem sampling and determination of cambial activity

Seasonal wood and phloem development was monitored during the growing seasons of 2011 and 2012 by taking small punched cores from eight trees/species of the outermost tree rings (micro-cores) with a diameter and length of 2.5 mm and *c.* 2 cm, respectively (Rossi et al. 2006a). To determine the variability in intra-annual wood and phloem formation between trees, individual trees were randomly selected. However, trees with major stem or crown anomalies were excluded from the analysis. Micro-cores were taken from March to October in weekly to 10-day intervals to include the whole dynamics of xylem formation. Shorter time intervals were chosen at the beginning and end of the growing season to determine the onset and end of cambial activity and xylem differentiation more precisely.

Samples were taken on the slope-parallel side of the stem following a spiral trajectory up the stem starting at *c.* 1 m stem height. A distance of 2–3 cm in tangential and longitudinal direction was kept to avoid lateral influence of wound reactions on adjacent sampling positions. Micro-cores were sampled from the same dominant trees during both study years having mean age of *c*. 150 year (*P. sylvestris*, *L. decidua*) and *c*. 115 year (*P. abies*) and mean diameter of 26.5–31 cm at breast height (cf. Schuster and Oberhuber 2013a).

**Fig. 1:**
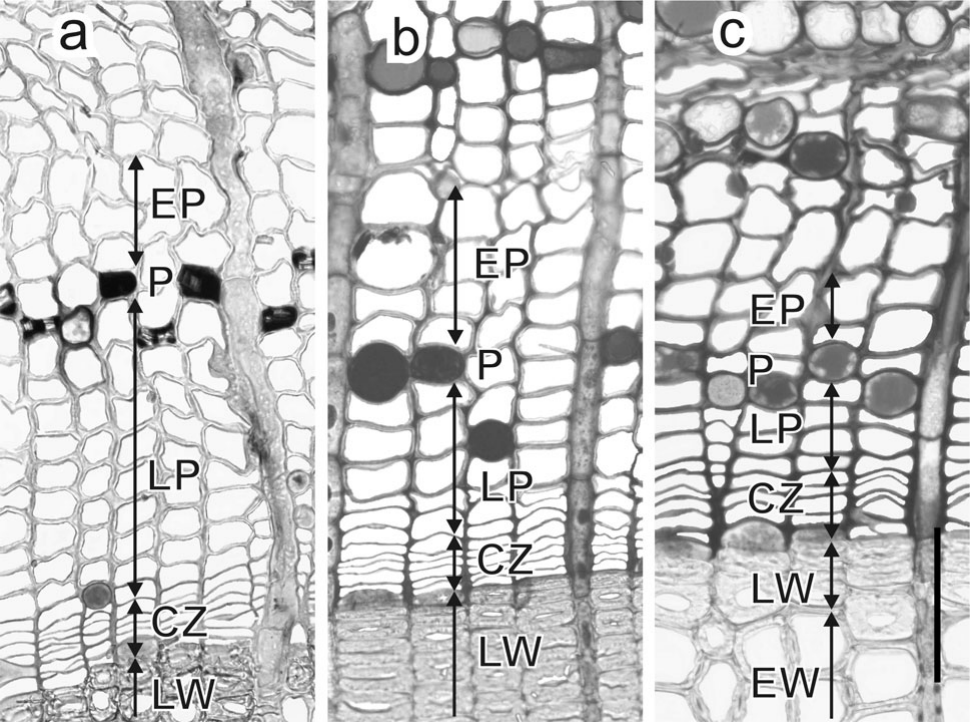
Cross-sections of the outer part of the stem in **a** *P. sylvestris*, **b** *P. abies* and **c** *L. decidua* after the growing season 2011 in early October showing secondary xylem, cambial zone and secondary phloem. Axial parenchyma cells with dark content separate early from late phloem cells (*EW* early wood tracheids, *LW* late wood tracheids, *CZ* cambial zone, *P* axial parenchyma, *LP* late phloem cells, *EP* early phloem cells). *Scale bar* 100 µm

Immediately after extraction, cores were stored in a microtube with 10 % aqueous ethanol and stored at 5 °C. Subsequently, cores were embedded in glycolmethacrylate (Technovit 7100) and polymerized after adding an accelerator. Transverse sections of *c*. 12 µm were cut with a rotary microtome, stained with a water solution of 0.05 % cresyl fast violet and observed under a light microscope with polarized light to differentiate the development of xylem cells—i.e., the discrimination between tracheids in enlarging and cell wall thickening phase (Deslauriers et al. 2003; Rossi et al. 2006b). The number of cambial cells (i.e., fusiform cells lacking radial enlargement), radial-enlarging cells, cells undergoing secondary wall thickening and lignification, and mature xylem cells were counted on all sampled cores in three radial rows. Cells in the cambial zone had thin cell walls and small radial diameters. Cells in radial enlargement were larger than cambial cells and observations under polarized light discriminated between enlarging and birefringent wall-thickening tracheids. During secondary wall thickening, the walls of cells changed from light violet (unlignified secondary cell walls) to blue (lignified cell walls) and tracheids were considered mature when cell walls were completely blue (cf. Rossi et al. 2006b). In accordance with Gričar and Čufar (2008), we determined first phloem cells by their slightly rounded thin tangential walls and differentiated phloem cells into early and late phloem sieve cells, which were separated by an axial parenchyma band (Fig. 1a–c). Thinner cell walls and wider radial dimensions also helped to separate early from late phloem sieve cells. Due to partly heterogeneous phloem development along individual radial rows, the number of phloem cells was determined by taking the whole transverse section into consideration. Xylem and phloem formation were considered to have begun when one horizontal row of radially enlarging tracheids and differentiating sieve cells (i.e., early phloem cells) were detected, respectively. The shape of these newly differentiating tracheids and sieve cells is readily distinguished from the cambial zone by their larger lumina, while thicker cell walls characterize latewood and late phloem cells formed in the previous year. Throughout the growing season, sieve cells developed species-specific differences in form, size and cell wall structure which had to be taken into account. Total xylem cell number was determined by adding the number of cells in radial enlargement, cell wall thickening, and the number of mature xylem cells. Total phloem cell number included early and late phloem sieve cells and axial parenchyma cells. End of xylem and phloem formation was defined when Gompertz-modeled dynamics of cell production (see below) reached 95 % of final number of modeled cells.

### Standardization of cell number and fitting of xylem growth

Because cell number varies within the tree circumference and hence among different samples, standardization is required (Rossi et al. 2003). The total cell numbers of the previous tree and phloem ring were recorded in every sample and used for a cell number correction for each tree. Cell number in each *j* sample (i.e., micro-cores taken throughout the 2011 and 2012 growing seasons) and by each *i* phase (i.e., enlarging, wall thickening, and mature tracheids and early and late phloem cells) was corrected as follows:

$$nc_{ij} = n_{ij} \times n_{\text{m}} /n_{\text{s}}$$

where

*nc*_ij_: Corrected cell number,
*n*_*ij*_: Counted cell number,
*n*_m_: Mean cell number of previous ring of all *j* samples, and
*n*_s_: Cell number of previous ring for each *j* sample.

Short-term variation in total number of tracheids (sum of enlarging, wall thickening and mature cells) and phloem cells (sum of early and late sieve cells and axial parenchyma cells) was modeled with a Gompertz function using the nonlinear regression procedure included in the Origin software package (OriginLab Corporation, Northampton, MA, USA). The Gompertz function, which is an asymmetric sigmoid curve, i.e., it accelerates more quickly than it decelerates, proved its versatility in describing growth-limiting processes and assessing cell dynamics of tree-ring growth (e.g., Deslauriers and Morin 2005; Rossi et al. 2003, 2006c).

### Microclimate records

During the study period, air temperature and precipitation were collected automatically (ONSET, Pocasset, MA, USA) within the stand at *c*. 2 m height and above canopy at top of a scaffold at *c*. 18 m height, respectively. Mean daily air temperatures were calculated by averaging all measurements (48 values per day). In addition, volumetric soil water content (ThetaProbes Type ML2×, Delta-T, Cambridge, England) and soil temperature (HOBO, ONSET, Pocasset, MA, USA) were recorded in the top 5–10 cm soil depth within the study plot. Measuring intervals were set to 60 min and mean daily water content (vol.%) and soil temperature (°C) were calculated by averaging all measurements (three sensors for each parameter).

### Environmental variables during growing seasons 2011 and 2012

**Fig. 2:**
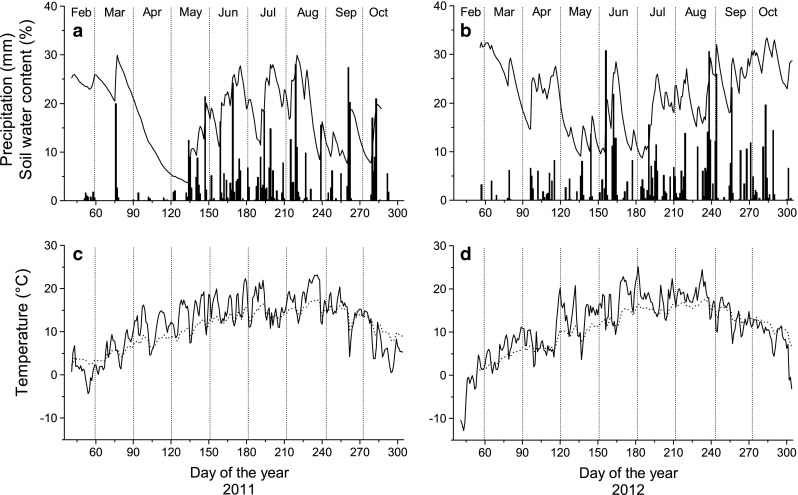
Climate variables and soil water content (vol.%) recorded during the growing seasons 2011 and 2012 within the study plot. **a**, **b** Daily precipitation sum (*bars*) and soil water content at 5–10 cm (*solid line*). **c**, **d** Mean daily air (*solid line*) and soil temperature (*dashed line*) recorded within the stand at 2 m height and in 5–10 cm soil depth, respectively

Climate in 2011 and 2012 distinctly deviated at the start of the growing season in spring. Most strikingly, mean daily air temperature in April 2011 amounting to 11.5 ± 3.2 °C was 3.2 °C higher compared to 2012 and an exceptionally drought period lasted from 19 March to 13 May 2011, which caused soil water content to drop below 10 vol.% from mid-April to mid-May 2011 (Fig. 2). Starting with rainfall events in mid-May 2011, soil moisture reached 20–30 vol.% until mid-August, when low rainfall caused again a decrease of soil moisture to *c*. 10 vol.% for several weeks. In 2012, more frequent rainfall events in April caused higher soil moisture content (*c*. 25 vol.%). The observed abrupt fluctuations in soil water content following precipitation events are caused by low water holding capacity of the shallow, stony soils. Daily mean soil temperature in 5–10 cm soil depth followed seasonal trends in air temperature and averaged 8.0 and 6.8 °C in April 2011 and 2012, respectively. On the other hand, air temperature and rainfall during summer 2012 amounted to 17.7 ± 3.3 °C and 364 mm, respectively, exceeding records in 2011 by 1.2 °C and 86 mm, respectively.

## Results

### Dynamics of tree-ring growth

**Fig. 3:**
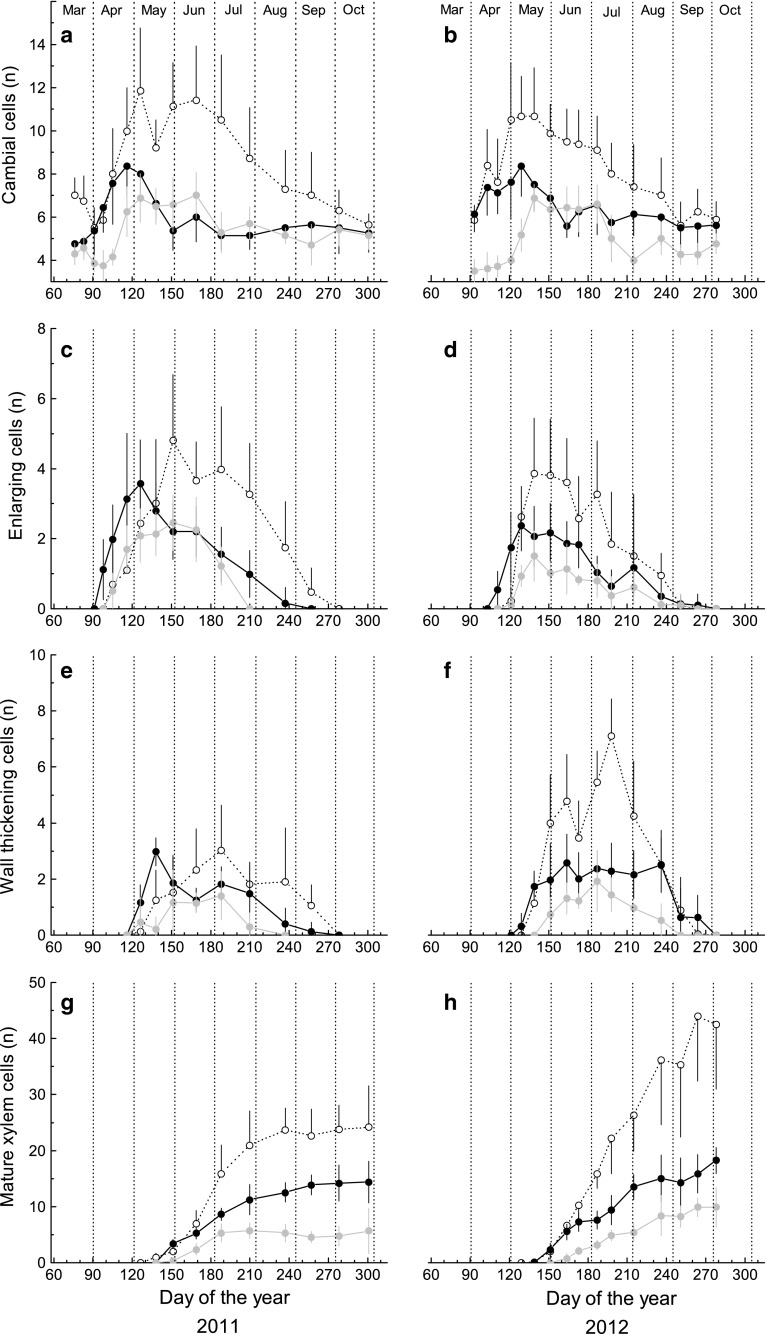
Number of cells **a**–**b** in the cambial zone, **c**–**d** in radial enlargement, **e**–**f** in secondary wall thickening and lignification, and **g**–**h** mature xylem cells during 2011 and 2012. Tracheid dynamics of different species are denoted by *open circle* and *dashed line* (*P. abies*), *closed circles* and *solid line* (*P. sylvestris*) and *closed grey circles* and *solid grey line* (*L. decidua*). *Bars* represent standard deviations and are partly plotted in one direction to avoid overlapping

The dormant cambium consisted of 5–6 cells in *P. sylvestris* and *L. decidua*, when there was no cambial activity from July through March. In *P. abies*, 6–7 cambial cells were developed during the dormant period lasting from August through March. During both growing seasons, the number of cambial cells reached highest values in *P. abies* amounting to 12 cells in June 2011. While cambial activity in *P. sylvestris* peaked in late April 2011 and early May 2012, a persistent cambial activity from May through June can be deduced in *L. decidua* and *P. abies* during both study years (Fig. 3a–b). Delayed bell-shaped curves (enlarging and wall thickening cells) and a growing S-shaped curve (mature cells) characterize the dynamics of tracheid differentiation (Fig. 3c–h). In 2011, onset of tracheid formation, i.e., detection of first enlarging cells, occurred in early April (100 doy) in *P. sylvestris* and in mid-April (111 doy) in *P. abies* and *L. decidua* (Table 1). In 2012, tracheid formation was delayed by *c*. 2 week compared to 2011. In both study years, onset of wall thickening occurred first in *P. sylvestris* in early through mid-May (128 ± 4 and 135 doy ± 5 in 2011 and 2012, respectively) and significantly delayed (*P* ≤ 0.05) in *P. abies* (144 ± 13 and 144 doy ± 6 in 2011 and 2012, respectively) and *L. decidua* (148 ± 17 and 156 doy ± 7 in 2011 and 2012, respectively). Wall thickening and lignification in *P.* *sylvestris* and *L. decidua* were completed about 3 week earlier in 2011 than 2012 (*P* ≤ 0.05), but did not differ significantly in *P. abies*.

**Table 1:** Onset, end, and overall duration of xylem (XylF) and phloem formation (PhlF) in *P. sylvestris*, *P. abies* and *L. decidua* in 2011 and 2012 (*n* = 5–8 trees/species)

	Onset XylF (doy)		End XylF (doy)		Duration XylF (days)		Onset PhlF (doy)		End PhlF (doy)		Duration PhlF (days)	
	2011	2012	2011	2012	2011	2012	2011	2012	2011	2012	2011	2012
*P. sylvestris*	100 ± 3^a^	116 ± 7^b^	237	234	137	118	81 ± 5^a^	95 ± 6^b^	263	250	183	155
*P. abies*	111 ± 6^b^	127 ± 4^c^	225	250	114	123	79 ± 4^a^	93 ± 0^b^	263	250	184	157
*L. decidua*	111 ± 6^b^	128 ± 3^c^	183	251	73	123	78 ± 6^a^	93 ± 0^b^	229	253	151	160

Timing of wood and phloem formation is given in days of the year (mean values ± standard deviation). Statistically significant differences of mean values between species (independent samples) and years (dependent samples) are indicated by different letters (*P* ≤ 0.05; Student’s *t* test)

**Fig. 4:**
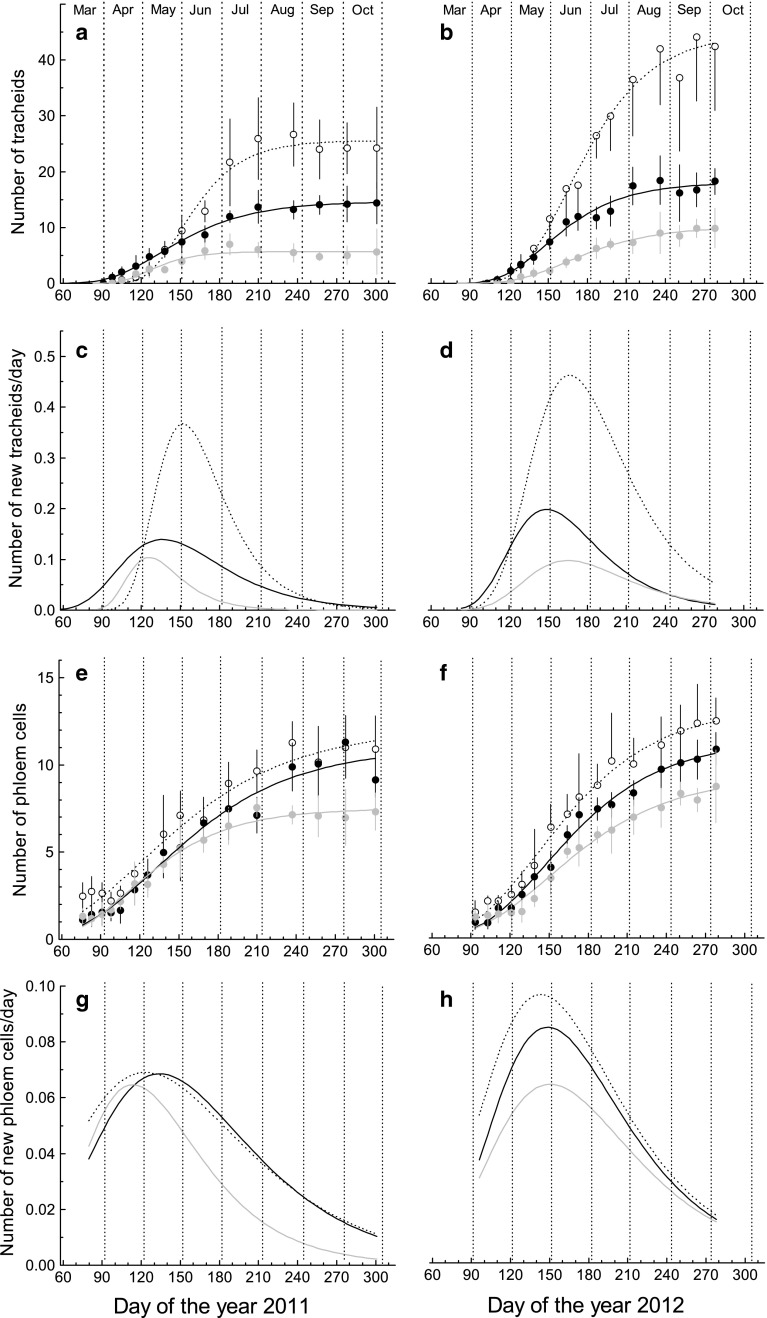
Dynamics of **a**–**b** xylem growth (including enlarging, wall thickening, and mature xylem cells) and **e**–**f** phloem growth (including early and late phloem and parenchyma cells) of all species modeled by applying the Gompertz function in 2011 and 2012. Daily growth rates of **c**–**d** xylem and **g**–**h** phloem calculated on the basis of modeled growth. Symbols and lines as in Fig. 3

A large variability in total number of tracheids between years and species was found (Fig. 4a–b). While in *L. decidua* only six and ten tracheids were developed in 2011 and 2012, respectively, *P. abies* produced 26 tracheids in 2011 and 45 tracheids in 2012. Based on modeled xylem cell number increase, cell production rate (tracheids d^−1^) culminated between early and late May in 2011 and between late May and mid-June in 2012 (Table 2). Cell production rate was highest in *P. abies* and lowest in *L. decidua* during both study years. When daily growth rates reached maximum values, cell production rate amounted to 5.6 and 1.4 cells within 14 days in *P. abies* and *L. decidua*, respectively (Fig. 4c–d). Mean duration of xylem formation during both study years lasted for about 4 months in all species, with the exception of quite lower growing season length in *L. decidua* in 2011, which was due to early ending of xylem formation in early June (Table 1). Xylem width amounted to 0.21 mm ± 0.1 (*L. decidua*), 0.35 mm ± 0.2 (*P. sylvestris*), and 0.76 mm ± 0.2 (*P. abies*) in 2011 and 0.36 mm ± 0.1 (*L. decidua*), 0.49 mm ± 0.2 (*P. sylvestris*), and 1.17 mm ± 0.3 (*P. abies*) in 2012.

**Table 2:** Parameters of the Gompertz function for xylem and phloem cell dynamics in 2011 and 2012 (see Fig. 4) of *P. sylvestris*, *P. abies* and *L. decidua* and *R* ^2^ of the model

Species	Year	Xylem formation				Phloem formation			
		A (*n* cells)	*I*_p_ (doy)	*κ*	*R* ^2^	A (*n* cells)	*I*_p_ (doy)	*κ*	*R* ^2^
*P. sylvestris*	2011	15 ± 0.4	134 ± 2.1	0.026 ± 0.002	0.993	11 ± 0.7	132 ± 6.3	0.017 ± 0.003	0.967
	2012	18 ± 0.7	147 ± 2.9	0.030 ± 0.004	0.980	11 ± 0.4	147 ± 2.8	0.020 ± 0.002	0.991
*P. abies*	2011	26 ± 0.9	150 ± 2.9	0.039 ± 0.006	0.981	12 ± 0.8	121 ± 6.4	0.015 ± 0.003	0.963
	2012	45 ± 1.9	165 ± 2.7	0.028 ± 0.003	0.988	13 ± 0.5	141 ± 2.9	0.020 ± 0.002	0.989
*L. decidua*	2011	6 ± 0.3	124 ± 4.0	0.050 ± 0.014	0.935	7 ± 0.2	112 ± 2.5	0.023 ± 0.002	0.983
	2012	10 ± 0.4	164 ± 2.4	0.026 ± 0.003	0.992	9 ± 0.5	149 ± 4.5	0.019 ± 0.002	0.981

Mean values ± standard deviation

*A* upper asymptote, *I*_p_ inflection point, *κ* rate of change parameter

### Dynamics of phloem growth

**Fig. 5:**
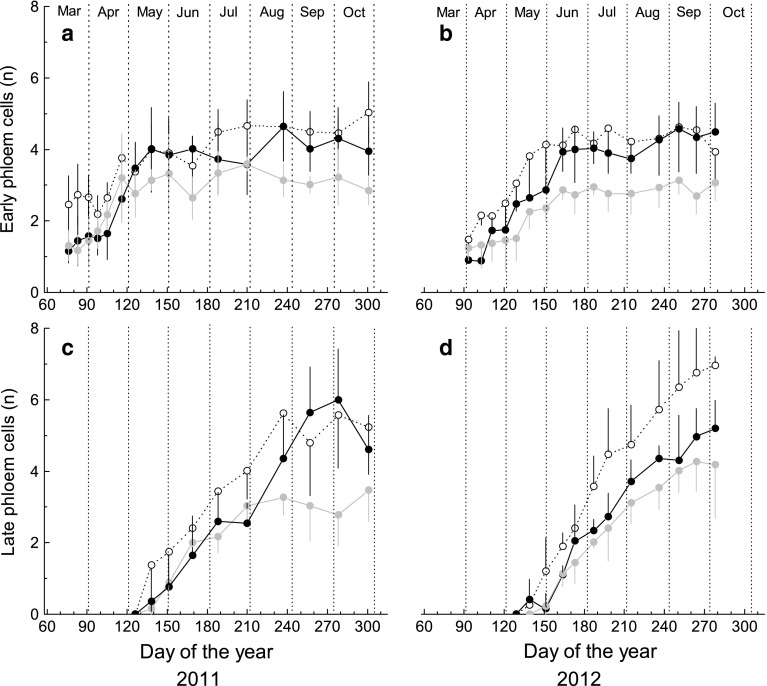
Number of cells **a**–**b** in early phloem, and **c**–**d** in late phloem during 2011 and 2012. Symbols and lines as in Fig. 3

Temporal formation of early and late phloem sieve cells in 2011 and 2012 is depicted in Fig. 5a–d. Onset of new phloem production, which was not significantly different among species in both years, occurred in mid-March 2011 and *c*. 2 week later in 2012 (*P* ≤ 0.05). Onset of phloem formation preceded xylem formation by 20 days in *P. sylvestris*, 33 days in *P. abies* and 34 days in *L. decidua* (mean values of 2011 and 2012; Table 1). Discontinuous tangential bands of parenchyma cells separating early phloem from late phloem sieve cells were developed around mid-May 2011 (doy 135 ± 12) in all species and in late May 2012 (doy 143 ± 6) in *P. sylvestris* and *P. abies* and in early June 2012 (doy 156 ± 7) in *L. decidua*. Correspondingly, continuous formation of late phloem cells started in mid-May 2011 and early June 2012. In 2011, ending of phloem cell formation, i.e., 95 % of total phloem cell number reached, occurred end of September in *P. sylvestris* and *P. abies*. Phloem formation of *L. decidua* in 2011 ended *c*. 4 week earlier, which is in agreement with earlier ending of xylem formation compared to other species (Table 1) and caused equally shortened duration of phloem formation. In 2012, phloem formation continued for about 5 months and ended in early September in all species.

**Fig. 6:**
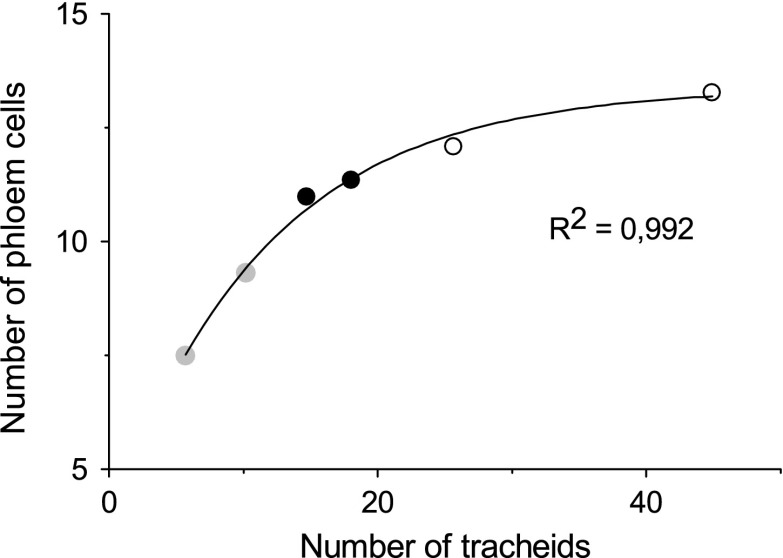
Relationship between number of phloem and xylem cells modeled by asymptotic regression (*y* = a − *b*.*c* ^*x*^). Symbols as in Fig. 3

Increase in total number of phloem cells (including early and late phloem sieve cells and parenchyma cells) during the growing seasons 2011 and 2012 is depicted in Fig. 4e–f. In contrast to large differences in number of xylem cells found among species, variability of modeled number of phloem cells was quite low ranging from 7–9 cells in *L. decidua* to 12–13 cells in *P. abies* (Table 2). Phloem formation culminated between late April and mid-May in 2011 and in late May 2012 (Fig. 4g–h; Table 2). In *P. sylvestris*, phloem and xylem formation peaked synchronously in both study years. On the other hand, phloem formation culminated *c*. 4 week and *c*. 2 week earlier than xylem formation in *P. abies* and *L. decidua*, respectively. Timing of maximum rate of xylem and phloem formation was delayed by *c*. 2 week in 2012 compared to 2011 in *P. sylvestris* and *P. abies* and by >5 week in *L. decidua* (Table 2). During study years, the mean ratio of xylem vs. phloem cells amounted to 1.0 in *L. decidua*, 1.5 in *P. sylvestris* and 2.8 in *P. abies* and was higher in 2012 compared to 2011 in all species. The relationship between total number of phloem and xylem cells was found to be curvilinear showing a gradual decrease in formation of phloem cells with increasing number of xylem cells (Fig. 6). Phloem width in 2011 amounted to 0.14 mm ± 0.04 (*L. decidua*), 0.23 mm ± 0.05 (*P. sylvestris*), and 0.21 mm ± 0.04 (*P. abies*) and in 2012 0.17 mm ± 0.05 (*L. decidua*), 0.25 mm ± 0.06 (*P. sylvestris*), and 0.27 mm ± 0.05 (*P. abies*).

## Discussion

### Seasonal dynamics of xylem formation

In temperate and boreal trees, temperature is known to be important for growth onset after winter dormancy (e.g., Linkosalo et al. 2006; Hänninen and Tanino 2011; Begum et al. 2013), and the existence of a temperature threshold above which significant tree growth in cold-adapted trees occurs has frequently been reported (for a review, see Körner 2006; Rossi et al. 2008). Therefore, significantly earlier onset of xylem and phloem formation by *c*. 2 week in 2011 compared to 2012 in all species is related to strikingly warmer temperature prevailing at the start of the growing season in 2011. Because an extended drought period lasted from late March through early May 2011, it is reasonable to assume that conifers drew upon stem water reserves mainly in elastic tissue of the bark and sapwood allowing temperature-induced growth resumption to occur (cf. Čermak et al. 2007). Statistically significant differences in onset of xylem formation might indicate that threshold temperatures for growth onset differ among species (Begum et al. 2013). In accordance with earlier onset of xylem and phloem formation in 2011, maximum daily growth rates peaked earlier in 2011 compared to 2012 in all species, which corroborates findings of Rossi et al. (2013) that the successive phenological phases of radial cell production are closely interconnected.

In accordance with previous findings reported by Gruber et al. (2010), xylem cell production in *P. sylvestris* culminated in May while in *P. abies* maximum rate of xylem formation peaked 2 week later during both study years, most likely indicating different life strategies of early and late successional species. Rathgeber et al. (2011) reported that social status can affect timing of cambial phenology, i.e., suppressed *Abies alba* trees started to grow later and had later maximum growth rate compared to dominant trees. Their findings might not apply to our study because all species occupy the dominant strata together. Furthermore, late successional *P. abies* presented strikingly higher tracheid production rates compared to early successional *P. sylvestris* and *L. decidua* indicating more efficient conversion of resources into radial growth in the former species. Minimal radial growth rates detected in *L. decidua* corroborate findings of Eilmann and Rigling (2012) and Lévesque et al. (2013) that *L. decidua* is maladjusted to dry conditions, which is possibly related to its anisohydric strategy, i.e., high transpiration rates are maintained even under drought (Leo et al. 2013). Because in *L. decidua* bud burst of short shoots occurred in early March 2011 (Swidrak et al. 2013), anisohydric behavior most likely caused quite early culmination of xylem formation in 2011, when water availability was strikingly reduced from late March through May. Earlier cessation of xylem formation in 2011 compared to 2012 in *P. abies* and *L. decidua* can be related to lower rainfall during summer, which is supported by findings that water deficits in summer cause early cessation of cambial activity in conifers within the study area (Pichler and Oberhuber 2007; Gruber et al. 2010) and in other drought-prone areas (e.g., Levanič et al. 2009; Thabeet et al. 2009; Eilmann et al. 2011).

### Seasonal dynamics of phloem formation

In all species and in both study years, onset of phloem formation considerably preceded that of xylem production, which is in accordance with previous findings by several authors (e.g., Alfieri et al. 1968; Davis and Evert 1968; Antonova and Stasova 2006, 2008; Gričar and Čufar 2008). Earlier cambial reactivation on the phloem side was also reported by Gričar et al. (2006) in artificially heated stems of *P. abies*. Early differentiation of phloem cells might be a prerequisite for wood formation to occur, because in temperate conifers all but the last-formed sieve cells cease functioning during the same growing season in which they are derived. Tree growth, however, requires a continuous supply of carbon as structural material and as a source for metabolic energy (e.g., Hacke et al. 2001). Furthermore, because accumulation of excess sugars in leaves leads to down-regulation of photosynthesis, assimilate transport from leaves is a necessary requirement for continuous photosynthetic production (Turgeon 2010; Nikinmaa et al. 2013), which in evergreen conifers commences at temperatures between −5 and −3 °C (Larcher 2003).

Temporal shift in onset and culmination of phloem cell formation in 2012 compared to 2011 in all species is in accordance with different timing of xylem cell production and related to delayed onset of tree growth in 2012, when quite lower temperatures prevailed at start of the growing season compared to 2011. While in *P. sylvestris* timing of daily maximum production of phloem and xylem cells was highly synchronized, culmination of phloem compared to xylem production occurred *c*. 2 week and *c*. 3 week earlier in *L. decidua* and *P. abies*, respectively. Inherent differences in endogenous control of phloem formation of coniferous species showing different sensitivity to drought and nutrient deficiency (Ellenberg and Leuschner 2010) could be an explanation for this finding.

Low variability in total number of phloem cells ranging from 7 to 13 cells was detected in all species and both study years indicating minor environmental and species-specific influence on phloem formation within the study area. The curvilinear relationship between number of phloem and xylem cells indicates an upper boundary of phloem cell production within the study area supporting endogenous control of phloem formation. The low ratio of xylem vs. phloem cells found in *P. sylvestris* and *L. decidua* is comparable to those reported by Gričar et al. (2009) for declining *Abies alba* trees. Several authors (e.g., Larson 1994; Plomion et al. 2001) suggested that the xylem–phloem ratio decreases under stressful environmental conditions, because phloem formation has priority over xylem formation due to restricted temporal integrity of sieve cells. Stressful environmental conditions within the study plot are indicated by narrow annual increments amounting to *c*. 0.4 mm in *P. sylvestris* and *c*. 0.3 mm in *L. decidua* and scattered occurrence of dead trees. On the other hand, slightly higher ratio of xylem vs. phloem cells in *P. abies* corresponds to considerably wider ring widths (*c*. 1.1 mm) indicating improved competitive status of late successional *P. abies* compared to early successional conifers. Schuster and Oberhuber (2013b) suggested that the *P. abies*’s competitive strength within the study area is related to efficient uptake of small rainfall events by its superficial root system and high water-use efficiency.

In summary, results of this study revealed that contrasting growth strategies are adopted by co-occurring early- and late successional species, which confirms our first hypothesis and is in accordance with results of Cuny et al. (2012) gathered in a temperate mixed coniferous forest in France. Regarding exogenous control of xylem formation, our second hypothesis could also be confirmed, because key phenological dates, i.e., onset and timing of maximum growth and total xylem increment showed distinct variability among study years, which were characterized by contrasting climate conditions. On the other hand, production of phloem cells was quite stable indicating tight endogenous control as expected. However, we have to partly reject our hypothesis that phloem formation is unresponsive to environmental factors, because in all species timing of onset and culmination of phloem formation was related to contrasting temperatures prevailing at the start of the growing season.
